# Diabetic Ketoalkalosis: A Case Report

**DOI:** 10.5811/cpcem.1389

**Published:** 2024-02-13

**Authors:** April Brill, Nirav Chheda, Daniel Strama, Ramesh Soundararajan

**Affiliations:** * Franciscan Health Olympia Fields, Emergency Department, Olympia Fields, Illinois; † Midwestern University Chicago College of Osteopathic Medicine, Downers Grove, Illinois

**Keywords:** *DKA*, *diabetic ketoalkalosis*, *baking soda ingestion*, *case report*

## Abstract

**Introduction:**

Diabetic ketoacidosis (DKA) is a common diagnosis in the emergency department (ED). However, one must consider other causes for acid-base disturbances when the pattern is not consistent with typical presentation.

**Case Report:**

A 52-year-old female with a history of insulin-dependent diabetes mellitus type 2 presented to the ED with abdominal pain, nausea, and vomiting for three days. Her diagnostic workup revealed diabetic ketoacidosis but with concurrent metabolic alkalosis. Standard treatment for DKA was initiated, and there was improvement of her mentation and resolution of metabolic derangements.

**Conclusion:**

Overlooking a diagnosis of DKA because of alkalosis on venous blood gas testing could lead to inappropriate treatment and, therefore, increased risk of morbidity and mortality in the affected patient.

Population Health Research Capsule What do we already know about this clinical entity?

*Diabetic ketoacidosis is a common diagnosis in the emergency department (ED), and at least 224,000 visits in 2016 were due to complications of hyperglycemia.*

What makes this presentation of disease reportable?

*This case demonstrates the setting of an altered patient who was found to be hyperglycemic but alkalotic secondary to exogenous ingestion of baking soda.*

What is the major learning point?

*This case discusses the importance of reviewing all lab values and the importance of fluids and electrolyte replacement as a standard of resuscitation.*

How might this improve emergency medicine practice?

*A thorough history and physical exam in cases of mixed acid-base disorders can provide pertinent information to help counsel patients on appropriate home management of diabetes and when to seek medical care.*

## INTRODUCTION

According to National Diabetes Statistics Report 2020, approximately 34.2 million people in the United States alone had been diagnosed with diabetes, which accounted for about 10% of the total population.^1^ Complications relating to hyperglycemic episodes accounted for 224,000 visits to emergency departments (ED) in 2016 alone.^1^ It has been estimated that the total cost of care related to diabetes has increased by 49 billion dollars between 2012–2017. Since diabetic complaints and complications are common, emergency physicians must be familiar with the disease process and sequelae.^1^ Morbidity and mortality due to diabetes and related disorders accounted for greater than 270,000 deaths in 2017 and was the seventh leading cause of death in the US in 2017.^1^ Here we describe a case of a 52-year-old female presenting with a unique cause of a mixed acid-base disorder for a diagnosis that is commonly encountered in the ED. We discuss in depth the evaluation of mixed acid-base disturbance. The case demonstrates the importance of obtaining a detailed history and physical exam whenever possible.

## CASE REPORT

A 52-year-old female presented to the ED by ambulance from home for evaluation of hyperglycemia. She reported complaints of abdominal pain, nausea, and vomiting over the prior three days. She noted that she administered her own medications, but the remainder of the history was limited due to encephalopathy. Further history was obtained from the paramedic report and electronic health record review because there was no family present at bedside. On chart review it was found that she had a past medical history of insulin-dependent diabetes type 2, gastroparesis, hypertension, seizure disorder, hyperlipidemia, depression, substance use (tobacco, cocaine, and marijuana), pancreatitis, and a hiatal hernia. Brief review of systems was significant only for nausea and vomiting, and she denied suicidality or use of drugs at that time. Surgical history was notable for pancreatectomy with partial autologous transplant and appendectomy. Reported home medications on the chart were as follows: amylase-lipase-protease 12,000–38,000–60,000 units delayed release capsule; sodium phosphate; di/mono and potassium phosphate monobasic 250 milligram (mg) tablet, aspirin 81 mg tablet, atorvastatin 40 mg tablet, calcium-vitamin D 500 mg-200 unit per tablet, docusate sodium 100 mg capsule, and insulin glargine U-100 100 units per milliliter (mL) vial.

On physical exam, vitals were temperature 36.4° Celsius, heart rate 80 beats per minute, respiratory rate 18 breaths per minute, blood pressure 135/72 millimeters mercury (mm Hg), oxygen saturation 97% on room air, and weight of 36 kilograms. She was ill-appearing and cachectic with dry mucous membranes, and her abdominal exam revealed focal epigastric tenderness without guarding, rebound, or rigidity. Her capillary refill and skin turgor were normal. She displayed no focal neurologic deficits and was alert and oriented to person, place, and time. However, her responses were tangential, and she displayed poor insight into her current situation and health problems.

Initial abnormal chemistry laboratory findings were sodium 126 milliequivalents per liter (mEq/L) (reference range 133–144 mEq/L), potassium 2.0 mEq/L (3.5–5.1 mEq/L), chloride 33 mEq/L (98–107 mEq/L), bicarbonate greater than 45 mEq/L (21–31 mEq/L), glucose 448 mg/deciliter (dL) (70–99 mg/dL), blood urea nitrogen (BUN) 75 mg/dL (7–25 mg/dL), creatinine 3.3 mg/dL (0.6–1.2 mg/dL), anion gap 48 mEq/L (6.2–14.7 mEq/L), and moderate acetone (negative). The mixed venous blood gas was also abnormal with a pH 7.64 (7.35–7.45); partial pressure of carbon dioxide 65.1 mm Hg (35–45 mm Hg); hemoglobin 11.9 grams (g)/dL (14–18 g/dL); and calculated bicarbonate 71.1 millimoles (mmol)/L (22–26 mmol/L). Serum alcohol was <10 mg/dL (<10 mg/dL). The full labs are listed in the Table. A chest radiograph revealed no acute cardiopulmonary findings, and a single view abdomen radiograph demonstrated a non-obstructive bowel gas pattern. Electrocardiogram was read as normal sinus rhythm, QTc 490 milliseconds, and no acute ST-T changes.

**Table.** Initial laboratory results with reference ranges in parenthesis of a patient with diabetic ketoalkalosis.

Complete blood count	Complete metabolic profile
WBC 5.8 (4.0–11.0 10^3^/uL)	Na 126 (133–144 mEq/L)
Hg 11.7 (12.0–15.3 g/dL)	K 2.0 (3.5–5.1 mEq /L)
Platelet 561 (150–450 10^3^/uL)	Cl 33 (98–107 mEq /L)CO_2_ >45.0 (21.0–31.0 mEq /L)
Acetone: moderate (negative)	Glucose 448 (70–99 mg/dL)
Lactic acid: 2.3 (0.5–2.0 mmol/L)	BUN 75 (7–25 mg/dL)Cr 3.3 (0.6–1.2 mg/dL)
Mixed venous blood gas:	Calcium 10.1 (8.6–10.3 mg/dL)
pH 7.647 (7.350–7.450)	Total protein 8.8 (6.4–8.9 g/dL)
PO_2_ 91.1 (80.0–95.0 mm Hgb)	Albumin 4.6 (3.5–5.7 g/dL)
PCO_2_ 65.1 (35.0–45.0 mm Hgb)	Total bilirubin 0.8 (0.0–1.0 mg/dL)
Hg 11.9 (14.0–18.0 g/dL)	Alkaline phosphatase 169 (34–104 U/L)
HCO_3_ 71.1 (22–26 mmol/L)	AST 37 (13–39 U/L)
	ALT 15 (7–52 U/L)
	Anion gap 48 (6.2–14.7 mEq/L)
	Mg 2.5 (1.6–2.6 mg/dL)
	Lipase 88 (11–82 U/L)
Urinalysis:	
Color: yellow (yellow)	Bilirubin: negative (negative)
Appearance: hazy (clear)	Protein 100 mg/dl (negative)
Ph 7 (5–8)	Blood urine: moderate (negative)
Specific gravity 1.010 (1.005–1.030)	Urobilinogen: negative (negative)
Glucose >500 mg/dl (negative)	Nitrite: negative (negative)
Ketones 20 mg/dl (negative)	Leukocyte esterase: negative (negative)
RBC 0–2 (0–2/hpf)	Bacteria: none seen (none seen)
WBC 0–5 (0–5/hpf)	Yeast: present (none seen)

*WBC*, white blood count; *μL*, microliter; *Hg*, hemoglobin; *g*, gram; *dL*, deciliter; *mmol*, millimole; *L*, liter; *PO_2_*, partial pressure of oxygen; *mm Hg*, millimeters of mercury; *PCO_2_*, partial pressure of carbon dioxide; *HCO_3_*, bicarbonate; *mg*, milligram; *hpf*, high power field; *RBC*, red blood cell; *NA*, sodium; *mEq*, milliequivalent; *K*, potassium; *CI*, chloride; *CO_2_*, carbon dioxide; *BUN*, blood urea nitrogen; *Cr*, creatinine; *U*, unit; *AST*, aspartate aminotransferase; *ALT*, alanine transaminase; *Mg*, magnesium.

Given the markedly abnormal metabolic derangements, initial treatment in the ED consisted of the following: two 1-L fluid boluses of 0.9 normal saline intravenous (IV); ondansetron 4 mg IV push, and potassium chloride (KCl) 60 mEq IV over six hours followed by normal saline with 20 mEq KCl at 150 mL per hour. There was concern for diabetic ketoacidosis (DKA) given the laboratory studies and a venous blood gas that demonstrated a mixed acid-base disorder with an elevated anion gap, moderate acetone, and elevated glucose. Insulin was initially ordered but cancelled when the potassium had not corrected enough prior to her admission to the intensive care unit (ICU). Due to the complexity of expected management of the metabolic derangements as well as acute kidney injury, critical care and nephrology were consulted to discuss further management.

During her stay in the ICU, she was eventually started on a continuous insulin infusion and dextrose 5% in water with 20 mEq KCl. Her anion gap closed, and her metabolic derangements resolved. She was switched back to her prior long-acting and sliding-scale insulin regimen. On day three of her stay, she was more cognizant and reported that she had been consuming baking soda at home for her symptoms prior to presentation. Once stabilized, she was downgraded to the general medical floor and discharged home on her previous medication regimen on hospital day six.

## DISCUSSION

The current literature reports on several cases of diabetic ketoalkalosis, a mixed metabolic acidosis and alkalosis disorder, in the setting of hypochloremia, although it remains a rare diagnosis.^2^ Metabolic alkalosis with hypochloremia can be secondary to excess vomiting, metabolic compensation, fasting or starvation state, or ingestion.^2^^–^^5^ We discuss the specific ingestion of baking soda as a cause for the patient’s metabolic derangements. Baking soda misuse has been shown to cause metabolic derangements resulting in hypokalemic metabolic alkalosis.^2^^,^^6^ Baking soda functions as an excess base with one teaspoon providing 59 mEq of bicarbonate compared to the 7.7 mEq found in a 650 mg tablet of sodium bicarbonate.^7^ Therefore, this ingestion also led to markedly decreased levels of chloride.^8^ Hypokalemic metabolic alkalosis due to excess baking soda consumption can occur even in patients with normal kidney function. However, this patient’s poor renal function, as indicated by her BUN and creatinine, further contributed to her inability to expel excess ingested bicarbonate, leading to an elevated level.^8^^,^^9^ This elevated level of bicarbonate along with the patient’s developing DKA resulted in a diabetic ketoalkalosis.

Besides the metabolic derangements, excess base ingestion can put patients at risk for dysrhythmias, seizures, and cardiopulmonary arrest.^9^ Treatment of the excess baking soda ingestion as well as the concomitant diabetic ketoalkalosis does not differ from that of DKA. After discontinuing use of the offending agent and initiating therapy with fluids, insulin, and potassium, all derangements normalized.^2^^,^^10^ It is significant to also discuss that insulin therapy is not recommended until hypokalemia is corrected to >3.3 mmol/L to decrease risk of arrhythmia and muscle weakness.^11^

The patient’s significantly elevated anion gap of 48 mEq/L confirms the metabolic acidosis, which was from DKA. Her severely elevated bicarbonate level in the chemistry panel of greater than 45 mqE/L, as well a blood gas with a calculated bicarbonate level of 71 mmol/L, confirms a significant metabolic alkalosis. This can also be corroborated by her pH of 7.647. Without the calculated bicarbonate level available from a blood gas, the modified delta gap could be used to screen for a mixed metabolic acid-base disorder. The formula is Delta gap=Na^+^- Cl_-_ − 36.^12^ In this case the delta gap was +9 and signified presence of a concomitant metabolic alkalosis since the rise in the anion gap was less than the fall of the bicarbonate level.^12^

In the case of this patient, at the onset of her symptoms she may have had simple hyperglycemia that resulted in gastroparesis with nausea and vomiting. The home remedy of baking soda may have caused her to progress into DKA as well as delay her presentation to healthcare personnel. Patients must be counseled on the potential risks of home remedies that can raise bicarbonate levels. This is especially true of those considering an alkaline diet or consuming a sodium bicarbonate antacid as this can predispose patients to hypokalemic metabolic alkalosis.^8^^,^^13^

## CONCLUSION

This case highlights the need for a thorough history and physical exam in cases of mixed acid-base disorders, since the offending agent was not identified until hospital day three. Although her treatment algorithm would not have changed with this information, it would have provided pertinent information to help counsel the patient on appropriate home management of her diabetes and when to seek medical attention.
